# Internet-delivered attention bias modification training in individuals with social anxiety disorder - a double blind randomized controlled trial

**DOI:** 10.1186/1471-244X-12-66

**Published:** 2012-06-25

**Authors:** Per Carlbring, Maria Apelstrand, Helena Sehlin, Nader Amir, Andreas Rousseau, Stefan G Hofmann, Gerhard Andersson

**Affiliations:** 1 Department of Psychology, Umeå University, Umeå, Sweden; 2 Department of Behavioural Sciences and Learning, Linköping University, Linköping, Sweden; 3 San Diego State University, San Diego, USA; 4 Department of Psychology, Boston University, Boston, USA; 5 Department of Clinical Neuroscience, Psychiatry Section, Karolinska Institute, Stockholm, Sweden

**Keywords:** Social phobia, Social anxiety disorder, Attention, Treatment, Information processing

## Abstract

**Background:**

Computerized cognitive bias modification for social anxiety disorder has in several well conducted trials shown great promise with as many as 72% no longer fulfilling diagnostic criteria after a 4 week training program. To test if the same program can be transferred from a clinical setting to an internet delivered home based treatment the authors conducted a randomized, double-blind placebo-controlled trial.

**Methods:**

After a diagnostic interview 79 participants were randomized to one of two attention training programs using a probe detection task. In the active condition the participant was trained to direct attention away from threat, whereas in the placebo condition the probe appeared with equal frequency in the position of the threatening and neutral faces.

**Results:**

Results were analyzed on an intention-to-treat basis, including all randomized participants. Immediate and 4-month follow-up results revealed a significant time effect on all measured dimensions (social anxiety scales, general anxiety and depression levels, quality of life). However, there were no time x group interactions. The lack of differences in the two groups was also mirrored by the infinitesimal between group effect size both at post test and at 4-month follow-up.

**Conclusion:**

We conclude that computerized attention bias modification may need to be altered before dissemination for the Internet.

**Trial registration:**

ISRCTN01715124

## Background

A recent development in the treatment of anxiety disorders is attention bias modification [1], which derives from basic research on information processing of threat relevant stimuli in various conditions such as social anxiety disorder [2]. There is an extensive literature on the link between attention and anxiety [3] and this link appears to be causal [4,5]. Attentional bias for threat in social anxiety is commonly measured using the probe detection task [6-8]. In the probe detection task [9] (for a review, see)[10], participants see a pair of faces at two different spatial locations on a screen. One of the faces is threatening, the other face is neutral. After the offset of these faces, a probe appears replacing the threatening face (congruent presentation) or neutral face stimulus (incongruent presentation). Faster responses to detect probes replacing threat faces than probes replacing neutral faces is used as an index of attentional bias toward threat relevant information.

Moreover, there is evidence that successful treatment for social phobia may lead to a normalization of attention bias for threat [11]. This finding is consistent with the view that attention bias to threat-relevant information plays a role in the maintenance of social phobia. Based on these preliminary findings, MacLeod and colleagues [12] reported the first study to train attention and examine its effect on anxiety. These researchers found that participants in the Attend Threat condition showed faster response latencies for detecting probes following threat words than neutral words. Participants in the Attend Neutral condition showed the opposite pattern of results. Moreover, this training extended to word pairs containing novel threat-relevant words and was not confined to specifically trained word pairs. Finally, participants in the Attend Threat condition responded more negatively to the experimental stressor than did those in the Attend Neutral condition. Extending this research, investigators have examined the effect of attention training in clinical populations. Recent reviews [4,5,13] suggest that attention training can be effective in in reducing anxiety in clinical and non-clinical population.

In a meta-analysis on the effects of attention bias modification by Hakamata and coworkers [14], 10 randomized controlled trials were identified including 467 participants. The average between group effect size was d = 0.61 on anxiety measures, and effects could be established up to 4 months after treatment termination. However, effect sizes differed widely between the studies, but a strength in this research is the possibility to have an active placebo control condition [5].

Attention bias modification is a potentially effective computerized treatment that most often has been delivered in a laboratory setting with subclinical samples *e.g.*, [15]. The training can sometimes be as short as one session with a total of 128 stimulus pairings [16]. The treatment involves repeatedly redirecting attention away from socially relevant threat cues in order to induce preferential selective processing of neutral (non-threat) stimuli [17]. Up until recently few studies on attention bias modification have targeted clinical populations of people suffering from social anxiety. One exception is a study in which 44 participants with generalized social phobia were randomized to one of two computerized attention bias modification programs [17]. Both conditions used a combination of faces expressing neutral and/or disgust emotions. After practicing twice a week during 4 weeks – equaling approximately 160 minutes in total – half of the participants in the treatment group as compared to 14% in the placebo group no longer met criteria for social phobia. In addition, there were significant effects on all outcome measures such as lower levels of anxiety, emotional distress, depression and better functioning in work, social and family life. The training was made at a university clinic, which theoretically could be a barrier for help seeking since eye-contact and talking to authority figures is a core problem for some of the sufferers. Hence, if the treatment could be administered *via* the internet that could open the door for millions of people in need. Another study that also found positive outcomes was carried out by Schmidt and coworkers [18]. At termination, 72% of patients in the active treatment condition, relative to 11% of patients in the control condition, no longer met criteria for social phobia. The results were maintained at 4-month follow-up. Finally, McEvoy and Perini [19] have investigated whether or not supplementing cognitive behavioral group therapy (CBT) with attention training could potentiate greater changes. It was tentatively concluded that while supplementing CBT with attention training did not improve outcomes, increasing attention control during CBT was associated with symptom relief.

Another recent development in the treatment of social anxiety disorder is the possibility to deliver CBT over the Internet [20]. A large number of trials have found that CBT delivered over the Internet can be as effective as face-to-face CBT, even in direct comparison [21,22], and effects have been documented by at least four independent research groups in Australia [23,24], Spain [25], Sweden [26,27], and Switzerland [28,29]. Given the two recent developments and the fact that attention bias modification is delivered *via* computer we decided to investigate if this novel treatment could be delivered *via* the Internet with no physical contact with the study participants. This has been done once in a recent trial by Boettcher and co-workers [30]. In fact, they could not replicate the promising results from the American studies. The aim of the present study was to test if attention bias modification delivered *via* the Internet was superior to a placebo condition (random attention training) in a group of participants with diagnosed social anxiety disorder recruited from the community. We hypothesized that the treatment would be better than the control condition and that it would be possible to present attention bias modification *via* the Internet.

## Methods

### Participants and recruitment

The general procedure was similar to our previous randomized controlled trials of internet-delivered self-help for social anxiety disorder [27,30,31]. Participants were recruited by media advertisements during the winter of 2009. A web page was created which included an outline of the study as well as general information about social anxiety disorder, the good results of the attention modification program used in the USA [17,18], ethical issues, internet security and a description of the study personnel. Participants filled out an application form and a computerized screening battery consisting of the Social Phobia Screening Questionnaire (SPSQ; [32]), the self-rated version of the Montgomery and Åsberg Depression Rating Scale (MADRS–S; [33]), the remaining outcome measures (see instruments below), and a few additional questions regarding current and past treatments. To be included, participants had to meet the following criteria: (a) a DSM–IV diagnosis of social anxiety disorder according to the SPSQ; (b) scoring below 31 on the MADRS–S depression scale and below 4 on the suicide item of this scale (to prevent the inclusion of individuals in strong need of specialist consultation); (c) not undergoing any other psychological treatment during the study period; (d) if prescribed drugs for anxiety or depression, the dosage had to be constant for 2 months before the treatment onset and kept constant throughout the study; (e) being at least 18 years old; (f) living in Sweden; (g) having access to a computer with internet connection; (h) not having a having a significant vision impairment, (i) not admitting another serious or dominant disorder (*e.g.* psychosis, substance misuse) that could be expected to influence the outcome of the study; j) having a primary diagnosis of social anxiety disorder according to the Structured Clinical Interview for DSM–IV Axis I Disorders (SCID–I; [34]). The last criterion was evaluated by a telephone interview in which the diagnostic questions from the social anxiety disorder section of the SCID–I were posed. When a person failed to meet the inclusion criteria, an individual encrypted message was sent with advice on how and where to seek more appropriate help.

**Figure 1:**
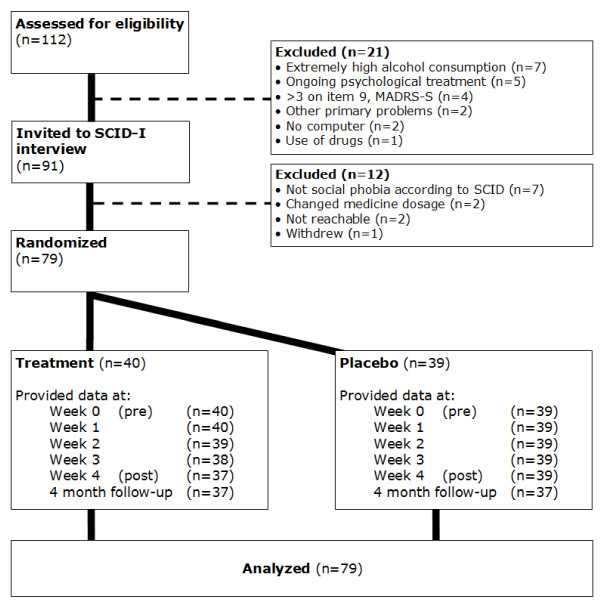
Flowchart of study participants, point of random assignment, and dropouts at each stage of a study of attention training in individuals with social phobia.

As evident from the CONSORT flowchart (Figure 1), of the 112 individuals who were assessed for eligibility 79 were subsequently included and randomized to attention modification training or a placebo group. Demographic data on the included participants are presented in Table 1.

**Table 1:** Demographic description of the participants at pre‒treatment

		**Treatment (n=40)**	**Placebo (n=39)**	**Total (n=79)**
Gender	Female	26 (65.0%)	28 (71.8%)	54 (68.4%)
	Male	14 (35.0%)	11 (28.2%)	25 (31.7%)
Age	Mean (SD)	35.1 (13.3)	38.0 (12.0)	36.5 (12.7)
Marital status	Married/living together	26 (65.0%)	18 (46.2%)	44 (55.7%)
	Single	11 (27.5%)	16 (41.0%)	27 (34.2%)
	Other	3 (7.5%)	5 (12.8%)	8 (10.1%)
Children	Mean (SD)	1.0 (1.0)	0.5 (0.8)	0.8 (1.0)
Registered sick	No	36 (90.0%)	36 (92.3%)	72 (91.1%)
	Yes	4 (10.0%)	3 (7.7%)	7 (8.9%)
Social phobia	Generalized	29 (72.5%)	30 (76.9%)	59 (74.7%)
	Non‒generalized	11 (27.5%)	9 (23.1%)	20 (25.3%)
Avoidant personality disorder (axis‒II)	Yes	14 (35.0%)	17 (43.6%)	31 (39.2%)
	No	26 (65.0%)	22 (56.4%)	48 (60.8%)
Medication	None	24 (60.0%)	21 (53.9%)	45 (57.0%)
	Earlier	8 (20.0%)	9 (23.1%)	17 (21.5%)
	Present	8 (20.0%)	9 (23.1%)	17 (21.5%)
Depression	Mean (SD)	14.6 (7.1%)	14.7 (6.4%)	14.7 (6.7%)
AUDIT	Mean (SD)	4.8 (3.5%)	4.3 (3.6%)	4.5 (3.5%)

Note: No significant differences existed between groups according to chi‒2 and independent t‒tests with Bonferroni correction for multiple tests.

### Measures

The following social anxiety scales constituted the outcome measures in the study: the Liebowitz Social Anxiety Scale self-report version (LSAS-SR; [35,36]), the Social Phobia Scale (SPS), the Social Interaction Anxiety Scale (SIAS; [35]), and the Social Phobia Screening Questionnaire (SPSQ; [32]). The LSAS-SR was the primary outcome measure. In addition, the following secondary measures were used to assess general anxiety and quality of life: the Beck Anxiety Inventory (BAI; [37]), and the Quality of Life Inventory (QOLI; [38]). In the screening phase the Montgomery Åsberg Depression Rating Scale (MADRS-SR; [33]) and the Alcohol Use Disorder Identification Test; AUDIT [39]) was used. The outcome measures have good psychometric properties even when administered *via* the Internet [40,41]).

### Treatment and placebo

Participants were either assigned to the real attention modification program or to a placebo version. Everything was identical in both conditions except for the location of the probe. Hence, in both conditions a trial began with a fixation cross (“+”) presented in the center of the screen for 500 ms. Immediately following termination of the fixation cue, the web based flash program in full screen mode presented two faces of the same person, one face on the top and one on the bottom, with each pair displaying one of two combinations of emotions. Either neutral-disgust, or neutral-neutral. After presentation of the faces for 500 ms, a probe appeared in the location of one of the two faces. Participants were instructed to indicate whether the probe was the letter E or F by pressing the corresponding arrow on the keyboard using their dominant hand. The probe remained on the screen until a response was given, after which the next trial began. During each session, 160 trials with various combinations of probe type (E/F), probe position (top/bottom), face type (neutral/disgust) and person (four male/four female were presented). There are a total of 8 persons showing 2 different facial expressions; 4 male and 4 female showing disgust or neutral. In the real condition the probe was always presented (100% of the trials) at the location of the neutral face if there also was a disgust face present (n = 128 trials). In contrast, in the placebo condition the location of the probe could not be predicted since the probe appeared with equal frequency in the position of the disgust face and the neutral face. The remaining 32 trials were neutral-neutral with the probe randomly presented at the top/bottom. For a more detailed description of the two conditions see Amir *et al.*[17]), since this study used the same procedures with the exception that the material was presented *via* the Internet.

Participants were encouraged to do the training on Tuesdays and Thursdays. They received an email and a SMS reminding them to do the training on the training days. If a session was missed a reminder was sent the following day. The participants could only do the training between 5 AM and 11 PM, and there should always be least one day between the sessions.

### Procedure and design

The participants were divided into two groups; treatment or control by an online true random-number service independent of the investigators. The study protocol was approved by the regional ethics committee, and written informed consent was obtained from all participants by surface mail.

All self-report scales were administered before the start of the treatment. During the treatment LSAS-SR once a week (Sundays). Following the four weeks of training LSAS-SR, SPS, SIAS, SPSQ, MADRS-S and QOLI was readministered. Immediately following the training phase, a clinical global impression of improvement (CGI-I) was mapped on a 7-point scale CGI; [42]) after a telephone interview by a blind assessor who had no earlier contact with the participants and no knowledge of to which group they had been randomly allocated. Finally, to check if the effects were stable over time a follow-up was conducted 4 months after the post-treatment assessment, conducted online by the respective participant.

### Analysis

In accordance with the intention-to-treat principle, all participants were asked to complete post-treatment and follow-up assessments, regardless of how many training sessions they had completed. Independent t-tests and chi-2 were used to check if randomization had resulted in a balanced distribution across both conditions. As evident from Figure 1, not all randomized participants provided complete datasets. The lowest response rate was 94.9% (75/79) at 4 month follow-up. In order to handle missing data in an intention-to-treat approach, we used a mixed models approach with a first-order autoregressive covariance structure as suggested by Gueorguieva and Krystal [43]. The first-order autoregressive covariance structure has the property that observations on the same subject that are closer in time are more highly correlated than measurements at times that are farther apart. Effect sizes (Cohen’s d) were calculated with the estimated means from the mixed model and by the following formula for converting standard error to standard deviation: SD = SE*(sqrt(n)).

## Results

Most participants (74 of 79) completed all 8 training session for a mean of 7.8. Tables 1 and 2 show that randomization resulted in a balanced distribution across both conditions at pre-treatment. However, no interaction effects were identified suggesting that the treatment did not outperform the placebo condition. This is echoed in the between-group effect size for the main outcome measure (LSAS-SR: Cohen’s d = 0.07; CI95% -0.29 to 0.23). Furthermore, the non-significant planned pair wise comparisons suggested that the treatment was without added value when controlling for repeated measurements and spontaneous remission by the use of a control condition. However, with the exception of the Quality of Life Inventory there were significant overall time effects on all measures for both groups. But it should be noted that the within-group effect sizes were small (treatment *d* = 0.15; placebo *d* = 0.19).

**Table 2:** Immediate and four months follow‒up results with intention‒to‒treat analysis using mixedeffect model estimating means and (standard deviation) for all participants

	**Treatment (n=40)**		**Placebo (n=39)**		**Pairwise comparison**	**Time (F)**	**Interaction (F)**
	**M**	**(SD)**	**M**	**(SD)**			
Liebowitz Social Anxiety Scale, self‒report						6.7***	1.7
Week 0	73.8	(35.1)	73.0	(35.6)	NS		
Week 1	71.3	(35.1)	74.3	(35.6)	NS		
Week 2	67.4	(35.2)	66.4	(35.6)	NS		
Week 3	64.8	(35.4)	64.1	(35.6)	NS		
Week 4	66.0	(35.5)	60.5	(35.6)	NS		
4 months	68.6	(35.6)	66.1	(35.8)	NS		
Social Phobia Screening Questionnaire						32.9***	0.6
Pre	31.9	(14.6)	33.3	(14.8)	NS		
Post	26.7	(14.8)	26.9	(14.8)	NS		
4 months	29.2	(14.9)	28.5	(15.0)	NS		
Social Phobia Scale						10.1***	0.3
Pre	40.3	(25.4)	40.4	(25.7)	NS		
Post	35.9	(25.7)	34.2	(25.7)	NS		
4 months	36.6	(25.8)	35.4	(26.0)	NS		
Social Interaction Anxiety Scale						5.1**	0.3
Pre	50.6	(24.2)	53.1	(24.5)	NS		
Post	47.5	(24.5	48.7	(24.5)	NS		
4 months	47.6	(24.6)	49.0	(24.8)	NS		
Beck Anxiety Inventory						9.6***	0.5
Pre	17.7	(12.8)	17.2	(12.9)	NS		
Post	15.3	(13.0)	13.4	(12.9)	NS		
4 months	16.2	(13.0)	14.1	(13.1)	NS		
Quality of Life Inventory						2.0	0.2
Pre	0.8	(2.5)	0.4	(2.5)	NS		
Post	1.1	(2.5)	0.6	(2.5)	NS		
4 months	1.0	(2.5)	0.6	(2.5)	NS		

*** p<.001; ** p<.01.

### Clinical significance

The CGI-I rating at post-treatment (n = 79) showed the following non-significant results for the treatment group *vs.* placebo group: very much improved (5.0% *vs.* 2.6%), much improved (2.5% *vs.* 20.5%), small improvement (32.5% *vs.* 35.9%), unchanged (57.5% *vs.* 38.5%) and small deterioration (2.5% *vs.* 2.6%) (chi-2(4, N = 79) = 7.5; p = 0.112). In addition to the self-report measures and the blind CGI-I rating a SCID interview was conducted with the results that three (7.5%) in the treatment group and two (5.1%) in the placebo group no longer met DSM-IV criteria for social phobia.

### Treatment adherence

Of the 79 participants who commenced treatment 74 (93.7%) completed all 8 training sessions as scheduled. However, five (6.3%) dropped out after only finishing M = 4.8 (SD = 1.0) sessions.

An analysis of the proportion of correct trials revealed no difference between the groups. The overall average was M = 98.7% correct indications of the letters presented. In addition, there was no difference in the average response time per trial M = 733 ms (SD = 290 ms).

### Change in attention bias

To get a measure of attention bias we followed the suggested procedure from the previous Amir study [17]. That included first eliminating response latencies for inaccurate trials. This procedure resulted in the elimination of 1% of the trials. Change in attention bias was subsequently calculated by subtracting response latencies of trials where the probe preplaced a neutral face of a disgust-neutral face pair from trial where both faces were neutral. This bias has also been used by other investigator to index disengagement [44,45]. In addition, response latencies less than 50 ms and greater than 1200 ms were considered outliers and were also eliminated from the analysis. These ranges were determined based on the inspection of the data using box plots and resulted in eliminating 1% of the trials. We submitted the participants’ performance on the Attention Task during each session to a 2 × 8 (Group [treatment, control] × Time [session 1–8]) analysis of variance (ANOVA). This analysis did not reveal a significant interaction of Group X Time (F(7, 69) = 1.15, p = .327). However, when analyzing the change scores on LSAS-SR there was a significant, albeit weak, positive correlation, indicating that the higher bias a participant had at the end of the treatment the more that participant improved (r = .32, p < 0.006). We calculated the correlation between session 1 bias score and change in LSAS. This correlation was only significant in the active group (r = 0.32, p < 005 active; r = −0.14, p = 0.37 placebo group).

To assess whether participants remained blind to their respective experimental condition, we asked the participants at post treatment whether they thought they had received the active or the placebo intervention. Of the participants who provided responses (n = 76) 18.9% (7/37) in the treatment group and 28.2% (11/39) in the placebo condition thought they had been randomized to the treatment program (Chi2(1, N = 76) = 0.9, p = .42).

## Discussion

This was a double blind randomized controlled trial with the aim to transfer the promising results of the Amir *et al.*[17] and Schmidt *et al.*[18] studies to the Internet. Results did not confirm our hypothesis that Internet-delivered attention bias modification would be superior to a placebo condition. However, there was a significant time effect, but that is probably best explained by spontaneous remission since both the treatment and the placebo group displayed similar gains. In addition, the within group effect sizes were small. This is the second randomized trial not being able to replicate the initial promising results. However, the first non-replication by Boettcher and coworkers was not identical to the original studies since different stimuli were used [30]. Specifically, the two original studies used the emotional facial expressions from Matsumoto and Ekman [46], while Boettcher and colleges used the NimStim faces [47]. The procedure in the present study mirrored the Amir study exactly including the same faces, number and spacing of the trials, and the location and duration of the stimuli. One obvious difference is that participants in the present trial did the training in a home setting as opposed to a university clinic ([17,18]). When looking at the proportion of correctly identified probes there was no difference and the marginally higher reaction time, as compared to Amir *et al.*[17] is explained by how elimination of individual trials were defined. The present study only excluded 3 SD above the individual trial mean (apx 1600 ms), while Amir *et al.*[17] simply excluded values higher than 1200 ms. Moreover, the method of delivery (*i.e.*, internet based flash program) *vs.* personal computer delivery was a difference between current study and previous research.

An explanation of the null findings could be that this study also included participants with non-generalized social phobia. However, the mean scores on LSAS-SR were almost identical (73.4 in this trial compared to 74.5 in the Amir trial). In addition, when running the analysis with the NGSP and GSP groups separated, no differences in the treatment effect emerged. 

When the participants who received the real treatment where asked to predict condition the absolute majority (81%) thought they had been randomized to the placebo group. One would think that such a low level of positive expectation would influence the trial. However, that cannot explain the lack of effect since the Amir *et al.*[17] and Schmidt *et al.*[18] papers reported almost identical numbers (78% and 94% respectively). Hence, there is a problem with the rationale and believability (*cf*.)[48]. However, the results when delivered on site have been impressive. Possibly attention training at home is negatively influenced by the fact that the person is sitting calmly in the comfort of his or her home. Perhaps there is an interaction between the active attention training and the mild anxiety that participants with social anxiety probably feel when they come to a university clinic to do their training (*cf*.)[49]. Although we instructed the participants to schedule the training when there would not be disturbed, there is a possibility that short breaks or non-focus reduces the effectiveness. Hence, the control over the procedures and the setting before, during and after training was suboptimal as compared to a lab. There could of course also be cultural differences. For example, in the case of panic disorder applied relaxation seems to work well in Sweden, but maybe not in the United Kingdom and USA [50,51]. The bias measure used in this study can be criticized. It could be argued that a task, in which the probe sometimes appears in the position of the disgust face and sometimes in the position of the neutral face, would be more accurate. However, Koster and coworkers [52] proposed that the probe detection task may be modified such that vigilance for threat and disengagement from threat may be assessed by including baseline trials, *i.e.*, trial with two neutral faces. Using this new measure of bias, Koster *et al.*, found that individuals with anxiety have had difficulty in disengaging their attention from highly threatening pictures. This measure of bias has been used but other investigators to assess the specific componets of attentional bias in anxiety [53-55]. The largest limitation is that participants were included without checking if they indeed had an initial attentional bias [1]. This is a serious flaw since not all patients with presenting with anxiety show bias [5]. However, the presence of a bias was not an inclusion criterion in the Amir *et al.*[17] or the Schmidt *et al.*[18] studies. Since there were no interaction effects in terms of change in bias between the two groups it can be concluded that not only was the internet-based treatment in the present study unable to modify attentional bias. In fact, there was the unexpected finding that larger bias was correlated with higher improvement. However, it should be noted that this was only a weak association, and could very well be explained as a random replicable finding. Indeed, as Heeren and coworkers have shown, engagement towards non-threat faces does not account for the positive treatment effects [56]. It should be noted that the intervention employed only trains attention away from negativity. There could be added benefits of instead of training what is essentially avoidance, to also or instead train active selection of positive mood-supporting information [57]. Perhaps the placebo condition, with equal frequency in the position of the threatening and neutral faces, is all that is needed to accomplish this disengagement from threat. Hence, future studies could, if done in an ethical way, test to include a condition of negative training.

## Conclusions

We conclude that attention bias modification may need to be further investigated before dissemination for the Internet.

## Competing interests

Seven of the eight authors declare that there is no conflict of interest. However, Dr Amir has founded a company that market online anxiety relief products.

## Authors’ contribution

All authors contributed to the design of this study. PC drafted the manuscript. All authors contributed to the further writing of the manuscript. All authors read and approved the final manuscript.
